# LncRNA LUESCC promotes esophageal squamous cell carcinoma by targeting the miR-6785-5p/NRSN2 axis

**DOI:** 10.1007/s00018-024-05172-9

**Published:** 2024-03-08

**Authors:** Song-tao Xue, Shi-qiang Cao, Jian-cheng Ding, Wen-juan Li, Guo-sheng Hu, Jian-cong Zheng, Xiao Lin, Chun Chen, Wen Liu, Bin Zheng

**Affiliations:** 1 Department of Thoracic Surgery, Fujian Medical University Union Hospital, No. 29 Xinquan Road, Fuzhou, 350001 Fujian China; 2 Key Laboratory of Cardio-Thoracic Surgery (Fujian Medical University), Fujian Province University, No. 29 Xinquan Road, Fuzhou, 350001 Fujian China; 3 State Key Laboratory of Cellular Stress Biology, School of Pharmaceutical Sciences, Faculty of Medicine and Life Sciences, Xiamen University, Xiang’an South Road, Xiamen, 361102 Fujian China; 4 Fujian Provincial Key Laboratory of Innovative Drug Target Research, School of Pharmaceutical Sciences, Faculty of Medicine and Life Sciences, Xiamen University, Xiang’an South Road, Xiamen, 361102 Fujian China

**Keywords:** ESCC, LUESCC, CeRNA, miR-6785-5p, NRSN2, ASO

## Abstract

Esophageal squamous cell carcinoma (ESCC) is one of the most prevalent gastrointestinal malignancies with high mortality worldwide. Emerging evidence indicates that long noncoding RNAs (lncRNAs) are involved in human cancers, including ESCC. However, the detailed mechanisms of lncRNAs in the regulation of ESCC progression remain incompletely understood. LUESCC was upregulated in ESCC tissues compared with adjacent normal tissues, which was associated with gender, deep invasion, lymph node metastasis, and poor prognosis of ESCC patients. LUESCC was mainly localized in the cytoplasm of ESCC cells. Knockdown of LUESCC inhibited cell proliferation, colony formation, migration, and invasion in vitro and suppressed tumor growth in vivo. Mechanistic investigation indicated that LUESCC functions as a ceRNA by sponging miR-6785-5p to enhance NRSN2 expression, which is critical for the malignant behaviors of ESCC. Furthermore, ASO targeting LUESCC substantially suppressed ESCC both in vitro and in vivo. Collectively, these data demonstrate that LUESCC may exerts its oncogenic role by sponging miR-6785-5p to promote NRSN2 expression in ESCC, providing a potential diagnostic marker and therapeutic target for ESCC patients.

**Supplementary Information:**

The online version contains supplementary material available at 10.1007/s00018-024-05172-9.

## Introduction

Esophageal cancer is the most prevalent malignant tumors in the digestive system, being the sixth leading cause of cancer-related death globally [1, 2]. China is one of those countries with the highest incidence of esophageal cancer [3]. Although significant progresses in the diagnosis and treatment of ESCC have been made in the past decade, ESCC is still largely incurable due to its poor prognosis and recurrence, with a five-year survival rate less than 30% [4]. Therefore, new diagnosis biomarkers and therapeutic approaches are urgently needed to improve the treatment of ESCC.

Recently, genome-wide studies have revealed the landscape of long non-coding RNAs (lncRNAs) with exquisite regulation of the malignant transformation via interaction with other cellular molecules at transcriptional, posttranscriptional, and/or translational level [5, 6]. LncRNAs are a group of transcripts longer than 200 nucleotides, which are transcribed from non-protein coding regions of the mammalian genome. They are important and powerful regulators of various biological activities and play a critical role in the progression of a variety of diseases, including cancer [5, 7]. Increasing evidences identify lncRNAs as tumor promotors or suppressors via affecting multitude cellular pathophysiological processes, highlighting their great potential in cancer diagnosis, prognosis, and therapy [8, 9]. MicroRNAs (miRNAs), a class of short non-coding RNAs with 19 to 25 nucleotides in length, are able to modulate both physiological and pathological processes by inducing mRNA degradation or translational inhibition through binding to the 3′-untranslated region (3′ UTR) of target protein-coding genes [10]. Currently, a model of lncRNA involvement in gene regulation called competing endogenous RNA (ceRNA) has been identified in which lncRNAs act as endogenous miRNA sponges to sequester them from binding to the 3′ UTR of target genes [11, 12]. For instance, high expression of lncRNA LINC00210 is detected in liver cancer and contributes to tumor progression by driving the activation of Wnt/β-catenin pathway in a CTNNBIP1-dependent manner [13]. LncRNA ESCCAL-1 exerted an oncogenic function in ESCC by positively regulating malignant behaviors of cancer cells during ESCC development via sponging miR-590-3p to modulate the expression of APOBEC3G [14]. LOC440173 was found to be upregulated in ESCC, which facilitates cell proliferation, migration, invasion, and epithelial-mesenchymal transition (EMT) process in vitro, and promotes tumor growth in vivo through competitively sponging miR-30d-5p to regulate HDX9 expression [15]. In our previous work, we have demonstrated an oncogenic lncRNA, LINC00680, promotes ESCC progression through the miR-423-5p/PAK6 axis [16]. Although more and more studies have demonstrated the critical roles of lncRNAs in ESCC, the underlying regulatory mechanisms remain incompletely understood.

Neurensin 2 (NRSN2), a small neuronal membrane protein that localized in small vesicles of neural cells [17], is found to be dysregulated in multiple types of human cancers. A previous study has revealed that NRSN2 is highly expressed in osteosarcoma tissues and promotes cell proliferation via the dysregulation of PI3K/Akt/mTOR and Wnt/β‑catenin signaling pathways [18]. NRSN2 is also closely associated with the malignant phenotype of ovarian cancer [19]. By contrast, it was demonstrated that NRSN2 is downregulated in hepatocellular carcinoma tissues and exerts a suppressive role during tumorigenesis [20]. These findings indicate that NRSN2 may function in a context-dependent manner.

Antisense oligonucleotides (ASOs) are short, single-stranded, and synthetic analogues of natural nucleic acids designed to specifically bind to the complementary RNA in a sequence-specific manner in both nucleus and cytosol of cells [21–23]. Importantly, ASOs can be designed to target genes associated with diseases including cancer [24–28], which makes ASOs as a kind of highly promising therapeutic strategy in clinics.

In this study, a lncRNA called LUESCC or RP11-160O5.1 (Refseq accession number: NR_110801) located on human chromosome 17q24.1 was found to be significantly upregulated in ESCC tissues, and predict a worse prognosis in ESCC patients. Functionally, knockdown of LUESCC repressed ESCC cell proliferation, colony formation, migration, and invasion in vitro and impaired tumor growth in vivo. Mechanistically, LUESCC sponged miR-6785-5p to enhance the expression of NRSN2, which promotes the malignant behaviors of ESCC. Of great clinical importance, interfering LUESCC by ASO was effective in suppressing tumor growth in mice. In summary, our data presented herein suggested that the LUESCC/miR-6785-5p/NRSN2 axis plays a pivotal role in ESCC progression, and LUESCC may serve as a diagnostic biomarker and therapeutic target for ESCC patients.

## Materials and methods

### Clinical tissue specimens

ESCC tumor tissues and adjacent normal tissues (at least 5 cm from the edge of cancer tissues) were obtained during surgical resection at the Fujian Medical University Union Hospital between 2015 and 2019. All clinicopathological diagnoses were confirmed by two experienced pathologists. None of the participants received preoperative radiotherapy or chemotherapy. After surgical resection, all specimens were placed in liquid nitrogen immediately and stored at –80 °C for further analysis. Informed consent was obtained from each patient. The study was granted by the Ethics Committee of the Fujian Medical University Union Hospital and performed according to the Declaration of Helsinki Principles.

### Cell culture

Human ESCC cell lines including KYSE140, KYSE150, KYSE510, EC109, and EC9706 as well as normal esophageal epithelial cell Het-1A were preserved by our laboratory for years and were maintained in RPMI 1640 (Biological Industries) medium supplemented with 10% fetal bovine serum (Biological Industries) and 1% penicillin/streptomycin mixture (Biological Industries). All cell lines were cultured at 37 °C in a humidified incubator containing 5% CO_2_.

### Cell transfection, lentivirus packaging and infection

siRNAs, miRNA mimics, miRNA inhibitors, ASOs, miRNA antagomirs, and matched negative control were obtained from RiboBio (Guangzhou, China). The same sequences of ASOs used in mouse xenograft models were modified by cholesterol at the 5′ end. The miRNA antagomirs are the chemically modified single-strand miRNA inhibitor with two phosphorothioates at the 5′ end, and four phosphorothioates and one cholesterol group at the 3′ end. All nucleotides are 2’-methoxy modified. To construct LUESCC expression vector, full-length LUESCC cDNA was synthesized and then inserted into the pBobi vector (Mailgene). To construct NRSN2 expression plasmid, the coding region of NRSN2 was cloned into the pCDH expression vector (Mailgene). Mock vector with no target sequence was used as a control. Short hairpin RNAs (shRNA) targeting LUESCC were synthesized (Bioray Biotechnology) and cloned into pLKO.1 vector. Oligonucleotide or plasmid transfections were performed using Lipofectamine 2000 or Lipofectamine™3000 reagent, respectively, according to the manufacturer’s instructions (Invitrogen). The sequences of oligonucleotides are listed in Table S1.

### RNA isolation, reverse transcription, and RT-qPCR

Total RNA was isolated by TRIzol reagent (Takara) according to the manufacturer’s description. RNA was reverse transcribed into cDNA using HiScript® II Q RT SuperMix (Vazyme). RT-qPCR was performed using Hieff® qPCR SYBR Green Master Mix (Yeasen) and AriaMx Real-Time PCR machine (Agilent Technologies). Actin and U6 were used as internal controls and all reactions were repeated in three independent experiments. The expression level was quantified with the 2^−ΔΔCt^ method. The primers used are listed in Table S1.

### RNA sequencing (RNA-seq) and computational analysis of RNA-seq data

ESCC cells KYSE510 and KYSE140 were treated with negative control siRNA or siRNA specifically targeting LUESCC or NRSN2 for three days, and then subjected to RNA-seq analysis. Total RNA isolation was performed using Trizol (Takara) followed by Dnase I digestion to remove residual DNA. RNA library preparation was performed using NEBNext® Ultra™ Directional RNA Library Prep Kit for Illumina (E7420L). Paired-end sequencing was performed with Illumina NovaSeq 6000. Sequencing reads were aligned to hg38 reference genome. Cufflinks was used to quantify the expression of RefSeq annotated genes with the option -M (reads aligned to repetitive regions were masked) and -u (multiple aligned read are corrected using ‘rescue method’) [29]. Genes with FPKM (fragments per kilobase per million mapped reads) larger than or equal to 0.5 in any one of the experimental conditions were included in our analysis. FPKM of a gene was calculated as mapped reads on exons divided by exonic length and the total number of mapped reads. DESeq2 was used to determine differentially expressed genes [30]. For differentially expressed genes in si LUESCC- or si NRSN2-transfected ESCC cells, a cutoff of *P* value less than 0.05 was applied. Box plot and heat map were generated by R software and significance was determined using Student’s t-test.

### Competitive endogenous RNA (ceRNA) network analysis

To construct ceRNA network, miRNAs that could bind to LUESCC were predicted by using three independent algorithms, miRanda (sequence align score, -sc 150) [31], RNAhybrid (minimal free energy, -e-23) [32], and TarPmiR (probability of target site, -p 0.8) [33], based on miRBase. mRNA targets that were demonstrated to be positively regulated by LUESCC were kept. The ceRNA network was constructed by Cytoscape [34].

### Cell proliferation and colony formation assay

Cell viability was assessed via MTS assay using CellTiter96®Aqueous One Solution Cell Proliferation Assay kit (Promega). Cells (3 × 10^3^/well) were inoculated in 96-well plates. After incubation for 24 h, 48 h, 72 h, and 96 h, the MTS solution (5 mg/ml, 10 μl) was added to each well mixed with 100 μl medium. Following incubation at 37 °C with 5% CO_2_ for 1 h, the absorbance at 490 nm was measured using a Multiskan MK3 Microplate Reader (Thermo Fisher, USA). For colony formation assay, cells were added in 6-well plates at a density of 2 × 10^3^ per well and incubated at 37 °C. Two weeks later, the cells were fixed with methanol and stained with 0.1% crystal violet, and the colonies were then counted.

### Cell migration and invasion assay

The migratory and invasive ability of transfected ESCC cells was assessed by wound healing and transwell assays, respectively. For wound healing assays, the transfected ESCC cells were cultured in serum-free medium and scratched using sterile 200 μl pipette tips at 80–90% confluence. The plate was washed three times with PBS to remove detached cells. At 0 h and 36 h of incubation, the images were recorded under a microscope (Carl Zeiss). The representative images were captured by an inverted microscope at 0 and 36 h after scratch. The quantification of the wound was calculated as the diminishing area across the induced injury area normalized to the 0 h control and expressed as a relative migration rate.

For invasion assay, transwell chamber harboring 50 ng/mL serum-free diluted Matrigel (Corning) was loaded onto the upper chamber according to manufacturer’s instructions. Total of 5 × 10^4^ cells resuspended in 300 µl serum-free medium was placed onto the upper chamber, while 500 µl medium containing 10% FBS was added to the lower chamber. After incubation for 24 h, cells remaining in the upper chamber were wiped off by a cotton swab and the invasive cells were fixed by methanol and following 0.1% crystal violet staining. The visualization of the crystal violet dyes was photographed and counted within three randomly chosen fields.

### Western blot assay

Total proteins were obtained from ESCC cells using RIPA extraction reagent (Beyotime) supplemented with PMSF protease inhibitor. The concentration was determined by the BCA method. The proteins were separated by SDS-PAGE, and then transferred to PVDF membranes (Millipore). The primary antibodies were incubated with PVDF membrane at room temperature for 2 h or at 4 °C overnight after blocking with skimmed milk. Subsequently, the membranes were exposed to the horseradish peroxidase-labeled secondary antibody for 2 h at room temperature and the chemiluminescent reagent (Millipore) was utilized to detect the protein bands. Primary antibodies against the following proteins were applied: NRSN2 (A14425, ABclonal), Lamin B1 (A11495, ABclonal), GAPDH (#5174, Cell Signaling Technology). Anti-rabbit IgG (#7074, Cell Signaling Technology) was employed as a secondary antibody.

### Subcellular fractionation

Nuclear and cytoplasmic fractions were prepared from ESCC cells cultured in 15 cm plates. Cells were washed twice with ice-cold PBS and then scraped gently into falcon tube (15 mL). Cell pellet was resuspended in 1 mL buffer containing 10 mM HEPES (pH 8.0), 1.5 μM MgCl_2_, 10 mM KCl, and 1 μM DTT, and then incubated for 15 min on ice to allow cells to swell. Suspension was added with 1% NP-40 and vortexed 10 s followed by centrifuge 2–3 min at 12,000 rpm. The supernatant was cytoplasmic fraction, whereas the pellet was nuclear fraction. Total RNA or protein was then extracted using Trizol (Takara) or RIPA (Beyotime) buffer according to the manufacturer’s guidance. The expression pattern of LUESCC in different cellular fractions was determined by RT-qPCR analysis with actin and U6 as the internal control for the cytoplasmic and nuclear RNA, respectively. The purity of cytosolic and nuclear fractions was further validated by immunoblotting analysis with anti-GAPDH and anti-Lamin B1 antibodies, respectively.

### Dual luciferase reporter assay

LUESCC or 3′-UTR of NRSN2 sequence containing the putative miR-6785-5p binding site as well as its mutant form were designed, synthesized and inserted into pmiR-RB-Report™ vectors (RiboBio), which were named as LUESCC (WT)-*luc*, NRSN2 (WT)-*luc*, LUESCC (MT)-*luc*, and NRSN2 (MT)-*luc*, respectively. The luciferase reporter (50 ng), negative control miRNA mimic (miR-NC) or miRNA mimic (40 nM) were co-transfected into ESCC cells seeded in 48-well plates. The firefly luciferase activity was determined 48 h after transfection with renilla luciferase activity as an internal reference using a dual luciferase reporter assay system (Promega, USA) in line with the manufacturer’s instructions.

### AGO2-CLASH

AGO2-CLASH assay was performed as previously described [35]. Briefly, ESCC cells plated in 10-cm petri dishes (70% confluence) are washed with PBS and cross-linked by ultraviolet (UV) exposure (50 mJ/cm^2^). Cross-linked cells are harvested and sonicated. AGO2 RISC complex is immunoprecipitated with anti-mouse AGO2 beads and washed. The RNAs in precipitates are phosphorylated using polynucleotide kinase (PNK), washed, eluted, extracted using phenol-isoamylalcohol and chloroform, precipitated with ethanol, washed with 70% ethanol, air dried, and dissolved in nuclease-free water. Finally, extracted RNAs are reverse-transcribed to single-stranded cDNA using HiScript® II Q RT SuperMix (Vazyme) and subjected to RT-qPCR analysis.

### Xenograft assays

Male BALB/c nude mice aged 4–5 weeks (18–20 g) were used for xenograft experiments and maintained under SPF conditions. All animal experiments were approved by the Animal Ethics Committee of Xiamen University.

For xenograft experiments, 5 × 10^6^ KYSE510 cells stably transfected with lentivirus vectors with shRNA against LUESCC or negative control were injected subcutaneously into the left flanks of nude mice (*n* = 5 per group).

For in vivo treatment with ASO and/or antagomir, 5 × 10^6^ wild type KYSE510 cells were inoculated subcutaneously into the left flanks of nude mice. One week later, nude mice were randomly divided into four groups (5 mice per group) when the tumor size reached approximately 80 mm^3^. ASO and antagomir alone or in combination were delivered by intratumor injection every 3 days at a dose of 5 nmol per injection (50 μL, 0.1 M in PBS) for five times. Tumor growth was monitored every 3 days with a vernier caliper. The tumor volume was calculated using the formula: 0.5 × length × width^2^. The mice were euthanized and tumor specimens were dissected, weighed and photographed at the end of experiments.

### Copy number detection

The copy number of LUESCC and miR-6785-5p in KYSE510 and KYSE140 cells was quantified by RT-qPCR method. In this assay, serially diluted RT-PCR products of LUESCC and miR-6785-5p were used as templates to formulate standard curves, and the exact copies of LUESCC and miR-6785-5p per cell were then calculated using the online tool (https://cels.uri.edu/gsc/cndna.html).

### Statistical analysis

All experimental data containing at least three independent repeats were presented as mean with standard deviation (SD) and investigated using GraphPad Prism 7.0. The comparison between two groups was assessed by Student’s t-test, while one-way analysis of variance (ANOVA) was utilized in multiple groups. Chi-square test was applied to evaluate the relationship between LUESCC expression and clinicopathological parameters. The survival curves were constructed with the Kaplan–Meier method and the log-rank test were performed for significance. Pearson’s correlation coefficients were employed to detect the expression correlation between different factors. All statistical analysis was performed using two-tailed *P*-values and *P*-value less than 0.05 was regarded to be statistical significance.

## Results

### A large number of lncRNAs are highly expressed in ESCC tissues and correlated with poor prognosis in ESCC patients

**Fig. 1:**
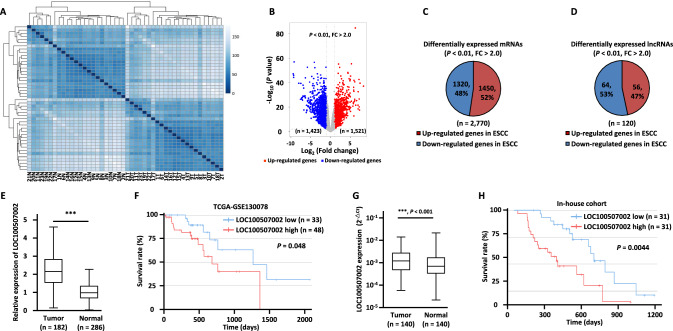
LncRNA LOC100507002 is over-presented in ESCC tumors samples, and predicts poor prognosis in ESCC patients.** A** The Hierarchical clustering analysis results for the 23 pairs of ESCC tumor tissues (T) and matched adjacent normal tissues (N) from GSE130078 are shown. **B** Volcano plot shows the differentially expressed genes in tumor and normal tissues as described in (**A**). Red and blue dots represent up- and downregulated genes in tumor samples, respectively (*P* < 0.01, FC > 2.0). **C**, **D** Pie chart shows the differentially expressed mRNAs (**C**) and lncRNAs (D) in ESCC tumor samples (*P* < 0.01, FC > 2.0). **E** The expression of LOC100507002 in a cohort of esophageal carcinoma (ESCA) samples (*n* = 182) and normal samples (*n* = 286) from GEPIA database. **F** Survival analyses of ESCC patients from TCGA cohort (GSE130078) based on the LOC100507002 expression level. **G** The expression of LOC100507002 in a cohort of 140 paired ESCC tumor and adjacent normal tissues in house is shown. **H** The correlation between the expression of LOC100507002 and the prognosis of patients for the ESCC cohort in house. Data were shown as mean ± SD, *P < 0.05, **P < 0.01, ***P < 0.001

To identify lncRNAs that are aberrantly expressed in ESCC, we analyzed the RNA sequencing data from The Cancer Genome Atlas (TCGA) (GSE130078) [36]. Hierarchical cluster analysis results indicated that the tumor samples could be well distinguished from the normal ones (Fig. 1A). Differential expression analysis results revealed that 1521 and 1423 Refseq genes were up- and downregulated in ESCC tumor samples compared to normal counterparts, respectively (*P* < 0.01, FC > 2.0) (Fig. 1B). Of the 2944 genes exhibiting greater than twofold differences in expression, 1450 mRNAs and 56 lncRNAs were upregulated, whereas 1320 mRNAs and 64 lncRNAs were downregulated in ESCC tumor samples (Fig. 1C, D). To identify lncRNAs that are clinically relevant, Kaplan–Meier survival analysis for all dysregulated lncRNAs (*n* = 120) were performed. The low expression of five lncRNAs, PLBD1-AS1, AGAP11, MAP4K3-DT, PLAC4, and LINC01206, predicted better prognosis, while the high expression of 14 lncRNAs, CD200R1L-AS1, KCNMB2-AS1, LOC101927136, BBOX1-AS1, CARMN, LINC00888, MIR4713HG, LINC00504, SLC44A3-AS1, LOC100507002, DLGAP1-AS1, RNF217-AS1, KLRK1-AS1, and ZNF503-AS1, was associated with poor prognosis (Table S2).

Among all the dysregulated lncRNAs that are clinically relevant, LOC100507002 (NR_110801.1), which has never been reported before, was selected for further investigation (Fig. 1E, F). To strengthen the clinical significance, LOC100507002 expression was remarkably elevated in ESCC tumor samples compared to adjacent normal tissues from 140 pairs of ESCC patients in-house (Fig. 1G). High expression of LOC100507002 was associated with poor prognosis in ESCC patients in our in-house cohort (Fig. 1H). Furthermore, multivariate analysis with clinicopathological information revealed that high expression of LOC100507002 was significantly correlated with gender, depth of invasion, and lymph node metastasis (Table S3). Consistently, we found that LOC100507002 presented at a much higher expression level in ESCC cell lines (KYSE140, KYSE150, KYSE510, ECA109, and EC9706) compared to normal esophageal epithelial cell line (Het-1A) (Fig. S1). Collectively, these results suggested that LOC100507002 is highly expressed in ESCC with clinical relevance. We renamed LOC100507002 as LncRNA Upregulated in Esophageal Squamous Cell Carcinoma (LUESCC).

### LUESCC promotes the malignant behaviors of ESCC

According to the UCSC Genome Browser annotation, LUESCC is located on human chromosome 17q24.1 with 700 base pair (bp) in length, and contains 3 exons (Fig. S2A). The transcript was confirmed to lack coding potential using CPAT dataset (http://lilab.research.bcm.edu/) and ribosome profiling (Fig. S2B, C) [37].

**Fig. 2:**
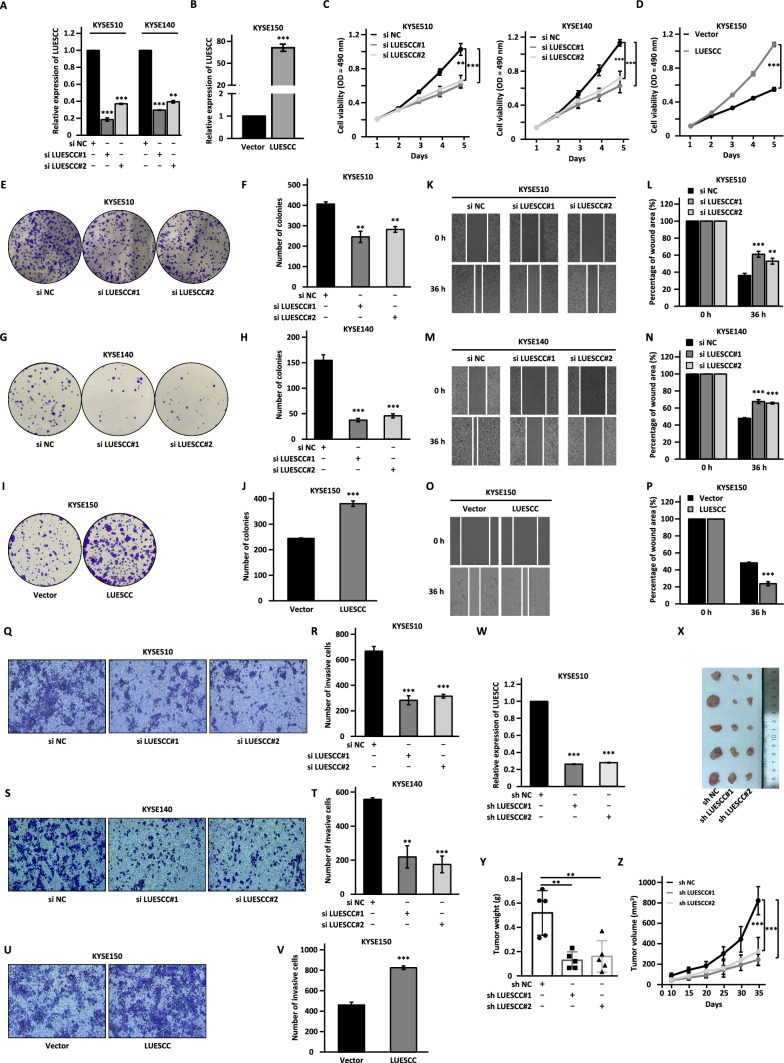
LUESCC promotes cell proliferation, colony formation, migration, and invasion in ESCC cells. **A** The expression of LUESCC in KYSE510 and KYSE140 cells transfected with control siRNA (si NC) or two independent siRNAs (si LUESCC#1 and si LUESCC#2) was examined by RT-qPCR. **B** The expression of LUESCC in KYSE150 cells transfected with control vector or vector expressing LUESCC was verified by RT-qPCR. **C–V** MTS (**C**, **D**), colony formation (**E**, **G**, **I**), wound healing (**K**, **M**, **O**), and transwell assays (**Q**, **S**, **U**) were performed in si LUESCC-transfected KYSE510 (**E**, **K**, **Q**) and KYSE140 (**G**, **M**, **S**) cells or LUESCC-transfected KYSE150 cells (**I**, **O**, **U**).** F**, **H**, **J**, **L**, **N**, **P**, **R**, **T**, **V** Quantification analysis results for colony formation (**F**, **H**, **J**), would healing (**L**, **N**, **P**), and transwell (**Q**, **S**, **U**) assays as shown in (**E**, **G**, **I**), (**K**, **M**, **O**), and (**Q**, **S**, **U**), respectively. **W** KYSE510 cells stably transfected with negative control shRNA (sh NC) or two independent shRNAs targeting LUESCC (sh LUESCC#1 and sh LUESCC#2) were subjected to RT-qPCR analysis. **X** Cells as described in **W** were injected subcutaneously into nude mice. Images of excised tumors are shown. **Y** The weight of tumors as described in **X** is shown. **Z** The growth curve of tumors as described in **X** is shown. Data were shown as mean ± SD, *P < 0.05, **P < 0.01, ***P < 0.001

To determine the biological function of LUESCC, control siRNA or two specific siRNAs targeting LUESCC were transfected into KYSE510 and KYSE140 cells followed by cell proliferation, colony formation, wound healing, and transwell assays. All these assays were also performed in KYSE150 cells transfected with control vector or vector expressing LUESCC (Fig. 2A, B). Knockdown of LUESCC led to a decline of cell proliferation, colony formation, migration, and invasion ability in KYSE510 and KYSE140 cells, while over-expression of LUESCC resulted in the opposite effects (Fig. 2C–V). To further assess the effects of LUESCC on ESCC tumorigenesis in vivo, KYSE510 cells stably transfected with control shRNA or two independent shRNAs targeting LUESCC were subcutaneously injected into nude mice (Fig. 2W). Tumors derived from sh LUESCC-infected KYSE510 cells grew much slower and were smaller in size than those derived from control cells (Fig. 2X–Z). Taken together, LUESCC promotes the malignant behaviors of ESCC.

### LUESCC is localized in the cytoplasm of cells and regulates the expression of NRSN2

**Fig. 3:**
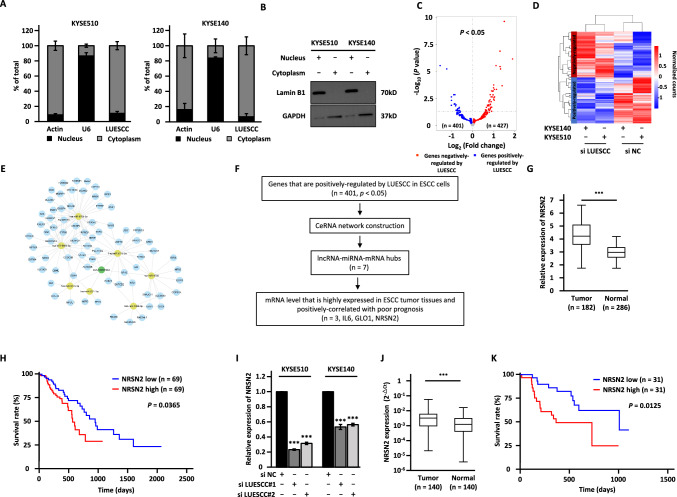
LUESCC regulates the expression of NRSN2, which is highly expressed in ESCC tumor samples and predicts poor prognosis in ESCC patients. **A** The subcellular distribution of LUESCC in KYSE510 (left panel) and KYSE140 (right panel) cells was determined by nuclear and cytoplasmic fractionation experiment followed by RT-qPCR analysis. **B** The nuclear and cytoplasmic fractionations as described in **A** were subjected to immunoblotting analysis. **C** KYSE510 and KYSE140 cells transfected with negative control siRNA (si NC) and siRNA specifically targeting LUESCC (si LUESCC) for three days were subjected to RNA-seq analysis, and differentially expressed genes are presented by volcano plot. Red and blue dots represent upregulated and downregulated genes, respectively, in both cell lines (*P* < 0.05). **D** The expression of differentially expressed genes (*P* < 0.05) as described in **C** is represented by heat map. **E** CeRNA network consists of LUESCC-miRNAs-mRNAs (genes positively regulated by LUESCC, *n* = 401) is shown. Nodes in green, yellow, and light blue represent LUESCC, miRNAs, and target mRNAs, respectively. **F** The flowchart to identify clinically relevant target genes of LUESCC in ESCC is shown. **G** The expression of NRSN2 in esophageal carcinoma tissues (*n* = 182) and normal tissues (*n* = 286) obtained from GEPIA database is shown. **H** The correlation between the expression of NRSN2 and prognosis of ESCA patients predicted by Oncolnc database is shown. **I** KYSE510 and KYSE140 cells transfected with negative control siRNA (si NC) or siRNA targeting LUESCC (si LUESCC#1 and si LUESCC#2) were subjected to RT-qPCR analysis to measure the expression of NRSN2. **J** The expression of NRSN2 in a cohort of ESCC tumor samples (*n* = 140) and normal samples (*n* = 140) in house is shown. **K** The correlation between the expression of NRSN2 and prognosis of ESCC patients as described in **J** is shown. Data were shown as mean ± SD, *P < 0.05, **P < 0.01, ***P < 0.001

We then investigate the molecular mechanisms underlying LUESCC regulation of ESCC tumorigenesis. The localization of lncRNAs in cells is closely related to their functions and mechanisms [38]. We, therefore, assessed the cellular localization of LUESCC by performing subcellular fractionation and RT-qPCR analysis. The results showed that LUESCC was largely distributed in the cytoplasm of ESCC cells (Fig. 3A). The purity of the cytosolic and nuclear fractions was further confirmed by immunoblotting analysis (Fig. 3B). Accumulating evidence has suggested that non-coding RNAs localized in the cytosol of cells may participate in ceRNA regulatory networks composing of lncRNA, miRNA, and mRNA [11]. We then explored the possibility that LUESCC functions as a ceRNA to sponge miRNA to regulate its target genes [39–42]. Transcriptome analysis was performed in KYSE510 and KYSE140 cells transfected with control siRNA or siRNA specifically targeting LUESCC to identify target genes regulated by LUESCC. Differential expression analysis results revealed that there were 401 and 427 genes positively and negatively regulated by LUESCC, respectively, in common in both cell lines (*P* < 0.05) (Fig. 3C, D). Three different algorithms (TarPmiR, miRanda, and RNAhybrid) were then utilized to predict potential miRNAs that can bind to LUESCC at high stringency, and then the highly confident miRNAs predicted were overlapped (Fig. S3A). Furthermore, lncRNA (LUESCC)-miRNA-mRNA network was constructed to connect LUESCC with its target genes and miRNAs. As depicted, seven miRNAs including miR-6883-5p, miR-6778-5p, miR-6785-5p, miR-4706, miR-2277-5p, miR-6778-3p, and miR-1908-5p, as well as 71 target genes were involved in the ceRNA network (Fig. 3E and Table S4). We then searched for target genes that are clinically relevant as LUESCC (i.e., highly expressing in ESCC tumor tissues and positively correlated with poor prognosis of ESCC patients) using GEPIA and Oncolnc webtool (http://www.oncolnc.org/), which led to the discovery of one gene named NRSN2 (Fig. 3F–H and Table S5). The effects of LUESCC knockdown on NRSN2 expression were further confirmed by RT-qPCR analysis (Fig. 3I). The high expression of NRSN2 in ESCC tissues and correlation with poor prognosis was independently confirmed using our in-house cohort (Fig. 3J, K). Furthermore, the correlation between the expression of LUESCC and NRSN2 was examined using TCGA database and our in-house cohort (Fig. S3B, C). Altogether, these results implied that LUESCC regulates the expressing of NRSN2, and NRSN2 might be a downstream target gene of LUESCC.

### LUESCC regulates the malignant phenotypes of ESCC cells through NRSN2

**Fig. 4:**
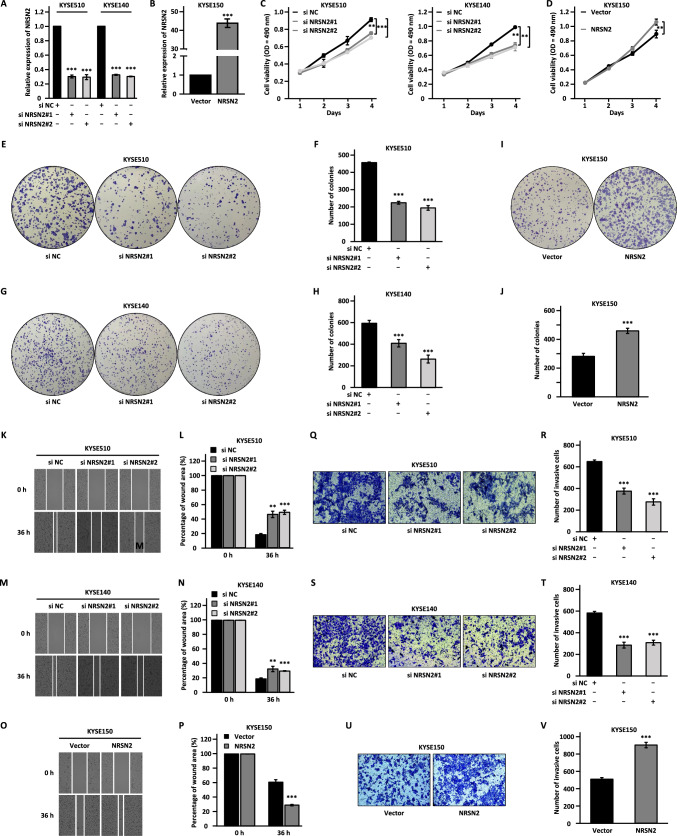
NRSN2 promotes the malignant behaviors of ESCC cells. **A** KYSE510 and KYSE140 cells transfected with negative control siRNA (si NC) or siRNA targeting NRSN2 (si NRSN2#1 and si NRSN2#2) were subjected to RT-qPCR analysis to examine the expression of NRSN2. **B** KYSE150 cells transfected with control vector or vector expressing NRSN2 were subjected to RT-qPCR analysis to examine the expression of NRSN2.** C–V** MTS (**C**, **D**), colony formation (**E**, **G**, **I**), wound healing (**K**, **M**, **O**), and transwell assays (**Q**, **S**, **U**) were performed in si NRSN2-transfected KYSE510 (**E**, **K**, **Q**) and KYSE140 (**G**, **M**, **S**) cells or NRSN2-transfected KYSE150 cells (**I**, **O**, **U**). **F**, **H**, **J**, **L**, **N**, **P**, **R**, **T**, **V** Quantification analysis results for colony formation (**F**, **H**, **J**), would healing (**L**, **N**, **P**), and transwell (**Q**, **S**, **U**) assays as shown in (**E**, **G**, **I**), (**K**, **M**, **O**), and (**Q**, **S**, **U**), respectively. Data were shown as mean ± SD, *P < 0.05, **P < 0.01, ***P < 0.001

We further explored whether NRSN2 is a downstream target gene, and whether LUESCC regulates the malignant behaviors of ESCC cells via NRSN2. To this end, we first examined the effects of NRSN2 on the malignant behaviors of ESCC cells. We transfected KYSE510 and KYSE140 cells with control siRNA or two siRNAs specifically targeting NRSN2, followed by cell proliferation, colony formation, wound healing, and transwell assays (Fig. 4A). Similar experiments were performed in KYSE150 cells transfected with vectors expressing NRSN2 (Fig. 4B). Knockdown of NRSN2 led to significant suppression on cell proliferation, colony formation, migration, and invasion, whereas NRSN2 overexpression had the opposite effects (Fig. 4C–V). These data indicated that NRSN2 might exerted a tumor-promoting effect in ESCC in vitro.

**Fig. 5:**
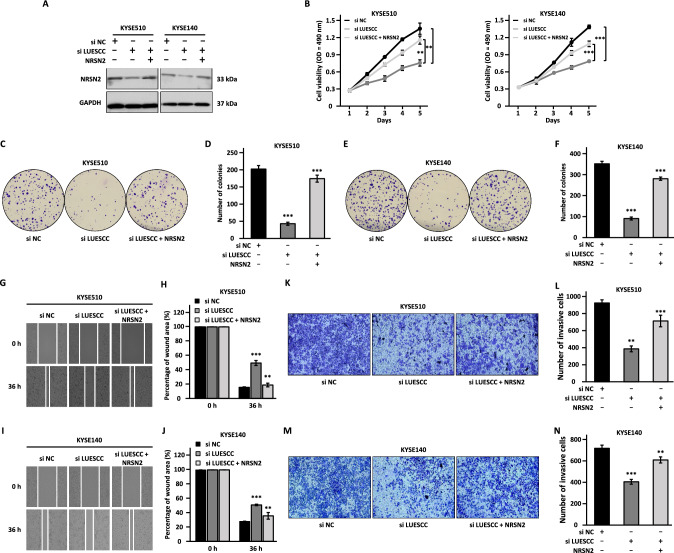
LUESCC regulates the malignant phenotypes of ESCC cells is partially dependent on the expression of NRSN2. **A–N** KYSE510 and KYSE140 cells were transfected with negative control siRNA (si NC) or siRNA targeting LUESCC (si LUESCC) in the presence or absence of vector expressing NRSN2 followed by western blot (**A**), MTS (**B**), colony formation (**C**, **E**), wound healing (**G**, **I**), and transwell assays (**K**, **M**). **D**, **F**, **H**, **J**, **L**, **N** Quantification analysis results for colony formation (**D**, **F**), would healing (**H**, **J**), and transwell assays (**L**, **N**) as shown in **C**, **E**, **G**, **I**, and **K**, **M**, respectively. Data were shown as mean ± SD, *P < 0.05, **P < 0.01, ***P < 0.001

To further test whether NRSN2 is a downstream target gene of LUESCC, rescue experiments were performed in KYSE510 and KYSE140 cells co-transfected with si LUESCC and NRSN2-expressing vector. The expression of NRSN2 was decreased upon LUESCC knockdown, which was restored when NRSN2 was overexpressed (Fig. 5A). Moreover, the inhibitory effects of LUESCC knockdown on cell proliferation, colony formation, migration, and invasion ability were attenuated by NRSN2 reintroduction (Fig. 5B–N). Thus, these results strongly suggested that LUESCC promotes ESCC progression, at least partially, through its target gene, NRSN2.

To understand the molecular mechanism underlying the LUESCC-NRSN2 axis in promoting ESCC progression, transcriptome analysis was performed in KYSE510 cells transfected with control siRNA or siRNA specifically targeting NRSN2. The results show that there are 2558 and 2208 genes positively and negatively regulated by NRSN2, respectively (FC > 1.5) (Fig. S4A, B, and Table S6). Gene ontology analysis results showed that calcium ion transport, regulation of blood pressure, and sensory perception of sound are among the top most enriched terms for genes positively regulated by NRSN2, and alpha-amino acid metabolic process, tRNA metabolic process, and dicarboxylic acid metabolic process are among the top most enriched terms for genes negatively regulated by NRSN2 (Fig. S4C, D). Furthermore, there were 411 or 343 genes were positively and negatively regulated by LUESCC and NRSN2 in common, respectively (Fig. S4E, F). In particular, TRPV6, ASIC2, MMP13, and FOXH1 are well known to be implicated in cancer development [43–47].

### LUESCC functions as a ceRNA to regulate NRSN2 and the malignant behaviors of ESCC cells via sponging miR-6785-5p

**Fig. 6:**
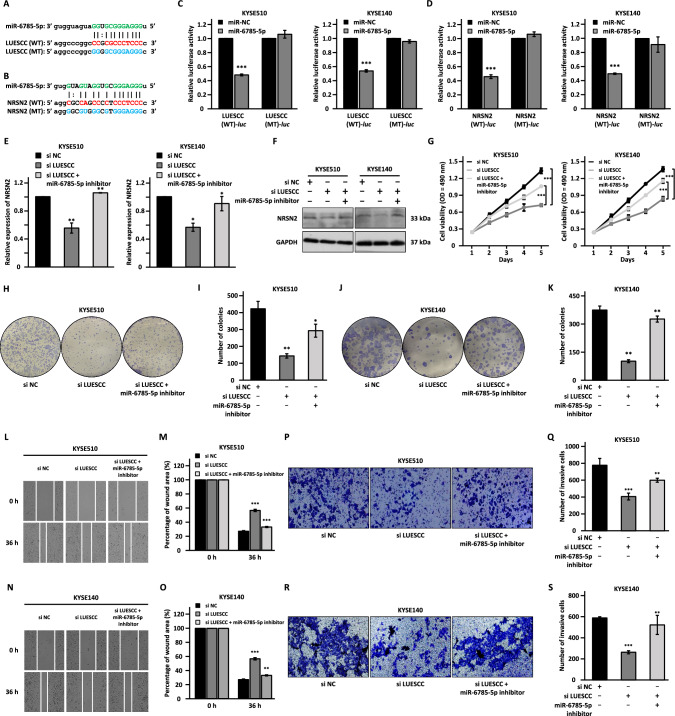
LUESCC acts as a miRNA sponge for miR-6785-5p to regulate the expression of NRSN2 and the malignant behaviors in ESCC cells. **A**, **B** Schematic representation of miR-6785-5p binding sites on wild-type (WT) LUESCC (**A**) and NRSN2-3′ UTR (**B**) as well as the corresponding mutant form (MT) with the predicted miR-6785-5p binding sites mutated. **C**, **D** KYSE510 and KYSE140 cells were transfected with reporters containing wild-type (WT-*luc*) or mutated (MT-*luc*) LUESCC (**C**) or NRSN2 3′ UTR (**D**) in the presence or absence of negative control miRNA mimic (miR-NC) or miR-6785-5p mimic (miR-6785-5p) followed by luciferase activity measurement. **E–S** KYSE510 and KYSE140 cells were transfected with negative control siRNA (si NC) or siRNA targeting LUESCC in the presence or absence of miR-6785-5p inhibitor followed by RT-qPCR (**E**), western blot (**F**), MTS (**G**), colony formation (**H**, **J**), wound healing (**L**, **N**), and transwell assays (**P**, **R**). **I**, **K**, **M**, **O**, **Q**, **S** Quantification analysis results for colony formation (**I**, **K**), would healing (**M**, **O**), and transwell assays (**Q**, **S**) as shown in **H**, **J**, **L**, **N**, and **P**, **R**, respectively. Data were shown as mean ± SD, *P < 0.05, **P < 0.01, ***P < 0.001

As shown by the ceRNA network, LUESCC might regulate NRSN2 expression via miR-6785-5p (Fig. 3E). Based on the starBase website, we found that miR-6785-5p could bind to the complementary sequence in LUESCC and the 3′ UTR of NRSN2 (Fig. 6A, B). DNA sequence containing miR-6785-5p recognition site in LUESCC, either wild type (WT-*luc*) or mutated form with miR-6785-5p recognition site mutated (MT-*luc*), was cloned into luciferase reporter vectors, which were then transfected into KYSE510 and KYSE140 cells together with control miRNA (miR-NC) or miR-6785-5p mimic followed by dual-luciferase reporter assay. The results showed that the overexpression of miR-6785-5p mimic led to a marked decrease in the luciferase activity of LUESCC (WT)-*luc* reporter compared with the miR-NC group, while no significant changes were observed for LUESCC (MT)-*luc* reporter (Fig. 6C). Similarly, the activity of NRSN2 (WT)-*luc* reporter was significantly decreased upon miR-6785-5p mimic transfection, while NRSN2 (MT)-*luc* reporter was not responsive (Fig. 6D). Furthermore, AGO2-CLASH assay results revealed the existence of LUESCC, NRSN2, and miR-6785-5p (Fig. S5A). To support that LUESCC could serve as a sponge for miR-6785-5p, the copy number analysis was performed (Fig. S5B, C). We found that the copy number of LUESCC and miR-6785-5p was approximately 496 and 153 copies per cell, respectively, in KYSE510 cells (Fig. S5D). Similar results were also obtained in KYSE140 cells, with that 514 and 245 copies were detected for LUESCC and miR-6785-5p, respectively (Fig. S5D). Moreover, miR-6785-5p expression was found to be downregulated in ESCC tissues and negatively correlated with that of LUESCC and NRSN2 (Fig. S5E–G).

To test whether LUESCC regulates the expression of NRSN2 and exerts its oncogenic role in ESCC is linked to miR-6785-5p, KYSE510 and KYSE140 cells were transfected with control siRNA or siRNA targeting LUESCC in the presence or absence of miR-6785-5p inhibitor. The expression of NRSN2 was suppressed in response to LUESCC knockdown both at mRNA and protein level, while this effect was obviously attenuated by miR-6785-5p inhibitor transfection (Fig. 6E, F). Moreover, miR-6785-5p inhibitor co-transfection partially abrogated the inhibitory effects of LUESCC silencing on cell proliferation, colony formation, migration, and invasion in both KYSE510 and KYSE140 cells (Fig. 6G–S). Collectively, LUESCC regulates the expression of NRSN2 and promotes the malignant phenotypes in ESCC cells through sponging miR-6785-5p.

### LUESCC is a potential therapeutic target for ESCC

**Fig. 7:**
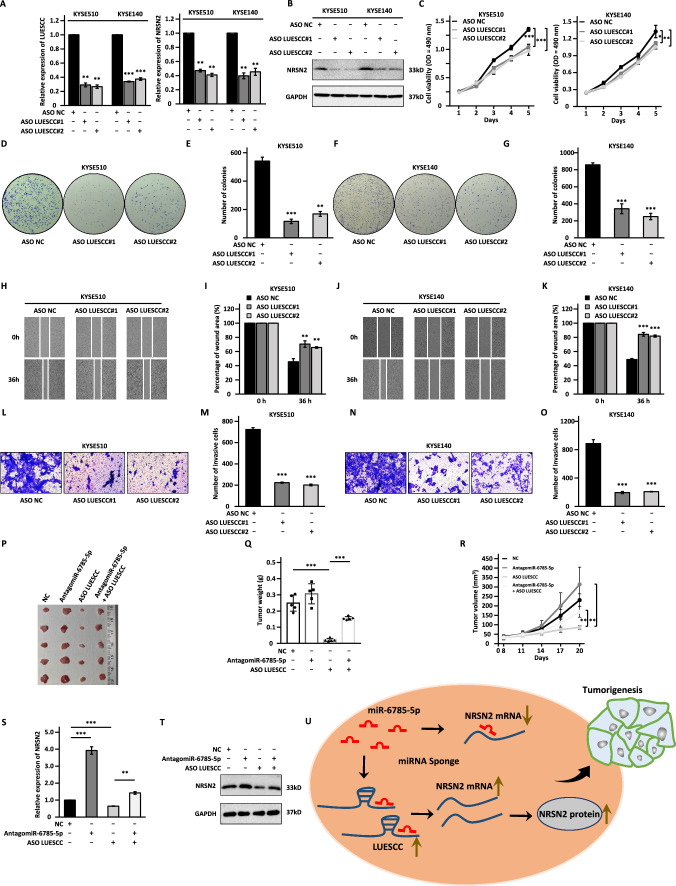
ASO specifically targeting LUESCC is effective in suppressing the malignant phenotypes of ESCC cells. **A**–**D**, **F**, **H**, **J**, **L**, **N** KYSE510 and KYSE140 cells were transfected with negative control ASO (ASO NC) or ASO specifically targeting LUESCC (ASO LUESCC#1 and ASO LUESCC#2) followed by RT-qPCR (**A**), immunoblotting analysis (**B**), MTS (**C**), colony formation (**D**, **F**), wound healing (**H**, **J**), and transwell assays (**L**, **N**). **E**, **G**, **I**, **K**, **M**, **O** Quantification analysis results for colony formation (**E**, **G**), would healing (**H**, **J**), and transwell assays (**L**, **N**) as shown in **D**, **F**, **H**, **J**, and **L**, **N**, respectively. **P** BALB/c nude mice were subcutaneously inoculated with KYSE510 cells and treated with or without ASO LUESCC in the presence or absence of antagomiR-6785-5p. Mice were then euthanized and tumors were collected. **Q**, **R** The weight (**Q**) and growth curve (**R**) of tumors as described in **P** are shown. **S**, **T** The expression of NRSN2 in tumors as described in **P** were examined by RT-qPCR (**S**) and immunoblotting (**T**) analysis. **U** A proposed model of LUESCC function in ESCC progression. The highly expressed LUESCC in ESCC cells functions as a miRNA sponge to sponge miR-6785-5p to release its repression on NRSN2 expression, leading to the aberrant expression of NRSN2 and tumorigenesis. Data were shown as mean ± SD, *P < 0.05, **P < 0.01, ***P < 0.001

To date, antisense oligonucleotide (ASO) drugs have attracted accumulating focus for their ability to specifically target and degrade target RNA both in vitro and in vivo [21, 28, 48, 49]. The upregulation of LUESCC in ESCC tumor samples, and its significant contribution to ESCC malignant phenotypes prompted us to exploit the possibility of LUESCC as a therapeutic target. For this purpose, two independent ASOs specifically targeting LUESCC (ASO LUESCC#1 and ASO LUESCC#2) and negative control (ASO NC) were synthesized and transfected into KYSE510 and KYSE140 cells. The mRNA expression of LUESCC was significantly inhibited by ASO compared to negative control group in both KYSE510 and KYSE140 cells (Fig. 7A). The expression of NRSN2, cell proliferation, colony formation, migration, and invasion ability were dramatically impaired upon LUESCC interference by ASO compared with negative control group (Fig. 7B–O). To further assess the in vivo anti-growth efficacy of ASO LUESCC and whether such effect can be linked to miR-6785-5p, BALB/c nude mice were subcutaneously inoculated with KYSE510 cells and treated with or without ASO LUESCC in the presence or absence of antagomiR-6785-5p. Compared to ASO NC-treated group, tumor weight and volume was significantly decreased in ASO LUESCC-treated group, which was largely restored by antagomiR-6785-5p treatment (Fig. 7P–R). To link LUESCC regulation of NRSN2 to ESCC tumorigenesis, significant reduction of NRSN2 was observed in tumors treated with ASO LUESCC compared to control group, which was notably restored in the presence of antagomiR-6785-5p (Fig. 7S, T). Taken together, the present results demonstrated that targeting LUESCC with ASO might serve as an effective therapeutic approach in ESCC.

## Discussion

The overall prognosis of ESCC remains unsatisfactory due to the lack of effective biomarkers for diagnosis and drugs for treatment [50]. To improve the survival rate of ESCC patients, a better understanding of the mechanism underlying ESCC progression is urgently required. LncRNAs have drawn increasing attentions for their important roles in the occurrence and development of human cancers including ESCC [51–54]. For example, the lncRNA MALAT1 [55] and HOTAIR [56] were overexpressed in ESCC tumor tissues and served as good predictive factors for overall survival. However, much is still unknown about the precise roles of lncRNAs in ESCC. A comprehensive understanding of the mechanisms of lncRNAs will contribute to the disclosure of the promising biomarkers and therapeutic targets for ESCC patients.

In the current study, we focused our attention on a functionally unknown lncRNA, LUESCC. We found that LUESCC was significantly upregulated in ESCC tissues, and was correlated with gender, depth of invasion, lymph node metastasis, and poor prognosis in ESCC patients. Functional experiments showed that knockdown of LUESCC inhibited ESCC cell proliferation, colony formation, migration, and invasion, while overexpression of LUESCC displayed the contrary effect. The tumor-promoting role of LUESCC was further demonstrated in vivo. These results give us the impetus to further exploit the biological mechanism of LUESCC in ESCC.

NRSN2 is a small neuronal membrane protein located in the small vesicles of neural cells [17]. Multiple studies have revealed the important roles of NRSN2 in the development of various types of cancers. NRSN2 overexpression was confirmed to be related with the malignant phenotype in osteosarcoma [18], ovarian cancer [19], and non-small cell lung cancer [57]. On the contrary, downregulation of NRSN2 was reported to serve as a suppressive gene in hepatocellular carcinoma [20]. In our study, NRSN2 was verified to be regulated by LUESCC, and was highly expressed in ESCC tumor samples. Furthermore, NRSN2 expression was positively associated with that of LUESCC. NRSN2 knockdown significantly inhibited cell proliferation, colony formation, migration, and invasion in ESCC, while overexpression of NRSN2 displayed the opposite effects. Importantly, the inhibitory effects of LUESCC silencing on ESCC malignant phenotypes were greatly abrogated by the overexpression of NRSN2. RNA-seq analysis results revealed that NRSN2 regulates the expression of genes implicated in calcium ion transport, regulation of blood pressure, sensory perception of sound, alpha-amino acid metabolic process, tRNA metabolic process, and dicarboxylic acid metabolic process. Dysregulation of the calcium channel, calcineurin/NFAT1, and TGF-β signaling pathway are well known to be implicated in cancer development [43–47]. From the above results, we concluded that LUESCC promoted ESCC progression, at least partially, by stimulating NRSN2 expression.

Multiple studies have demonstrated that some specific endogenous lncRNAs can act as ceRNAs to interfere with miRNA pathways, thereby alleviating their inhibition of target genes [11, 58]. The current results indicated that LUESCC was predominantly distributed in the cytoplasm of ESCC cells, implying that it may exert its effects via post-transcriptional regulation of mRNA targets through sponging miRNAs. The prediction results from ceRNA network analysis showed that miR-6785-5p could bind to both LUESCC and NRSN2, which was further confirmed by luciferase reporter and AGO2-CLASH assay. To date, miR-6785-5p had been proved to be involved in multiple malignant behaviors in previous studies [59–61]. In this regard, we further explored the role of miR-6785-5p in ESCC. In this study, miR-6785-5p was determined to be downregulated in ESCC tissues, and was inversely related with LUESCC and NRSN2 expression. Functionally, knockdown of LUESCC induced decreased expression of NRSN2, which could be partially reversed by miR-6785-5p inhibition. Furthermore, the anti-tumor effect of LUESCC knockdown was evidently reversed following the introduction of miR-6785-5p inhibitor in ESCC cells. These results indicated that LUESCC may accelerate ESCC progression by serving as a sponge for miR-6785-5p to relieve its inhibition on NRSN2 expression.

Current cancer treatment strategies using antibodies or small molecules pose several issues, such as severe toxic side effects, high dosage requirements, lack of tissue specificity, and unable to regulate pathogenic gene expression. In comparison, ASOs may offer an alternative approach to specifically target the underlying genetic cause of the cancer from RNA level to regulate the expression of critical pathogenic proteins dysregulated in cancer advancement [62]. Hence, the potential of LUESCC acts as a therapeutic target in lncRNA-based cancer therapy warrants further exploration. Here, we found that ASO targeting LUESCC substantially restrained cell proliferative, clonogenic, migratory, and invasive property in vitro and tumor growth ability in vivo. The evidence observed above supported that ASO targeting LUESCC may serve as a promising therapeutic method to retard LUESCC-induced ESCC carcinogenesis. Our results elucidated new insights into the dysregulated LUESCC/miR-6785-5p/NRSN2 regulatory axis in the development of ESCC, and unraveled potential diagnostic markers and therapeutic targets for ESCC.

In summary, we demonstrated that lncRNA LUESCC regulates NRSN2 expression via sponging miR-6785-5p to promote ESCC progression. LUESCC/miR-6785-5p/NRSN2 axis may serve as promising diagnostic and prognostic biomarkers, and therapeutic targets for ESCC patients (Fig. 7U).

### Supplementary Information

Below is the link to the electronic supplementary material.

Supplementary file1 (PPTX 1059 KB)

Supplementary file2 (XLSX 11 KB)

Supplementary file3 (XLSX 17 KB)

Supplementary file4 (XLSX 11 KB)

Supplementary file5 (XLSX 50 KB)

Supplementary file6 (XLSX 23 KB)

Supplementary file7 (XLSX 444 KB)

Supplementary file8 (DOCX 18 KB)

## Data Availability

All the data supporting the findings of this study are available within the article and its additional files, and from the corresponding author upon reasonable request.
