# Supplementary material for: LncRNA LUESCC promotes esophageal squamous cell carcinoma by targeting the miR-6785-5p/NRSN2 axis
Source: Cell Mol Life Sci. 2024 Mar 8;81(1):121. doi: 10.1007/s00018-024-05172-9 (PMC10924007; doi:10.1007/s00018-024-05172-9)
Supplement: Supplementary file 1 — Supplementary file1 (PPTX 1059 KB) [file 18_2024_5172_MOESM1_ESM.pptx]

## Slide 1
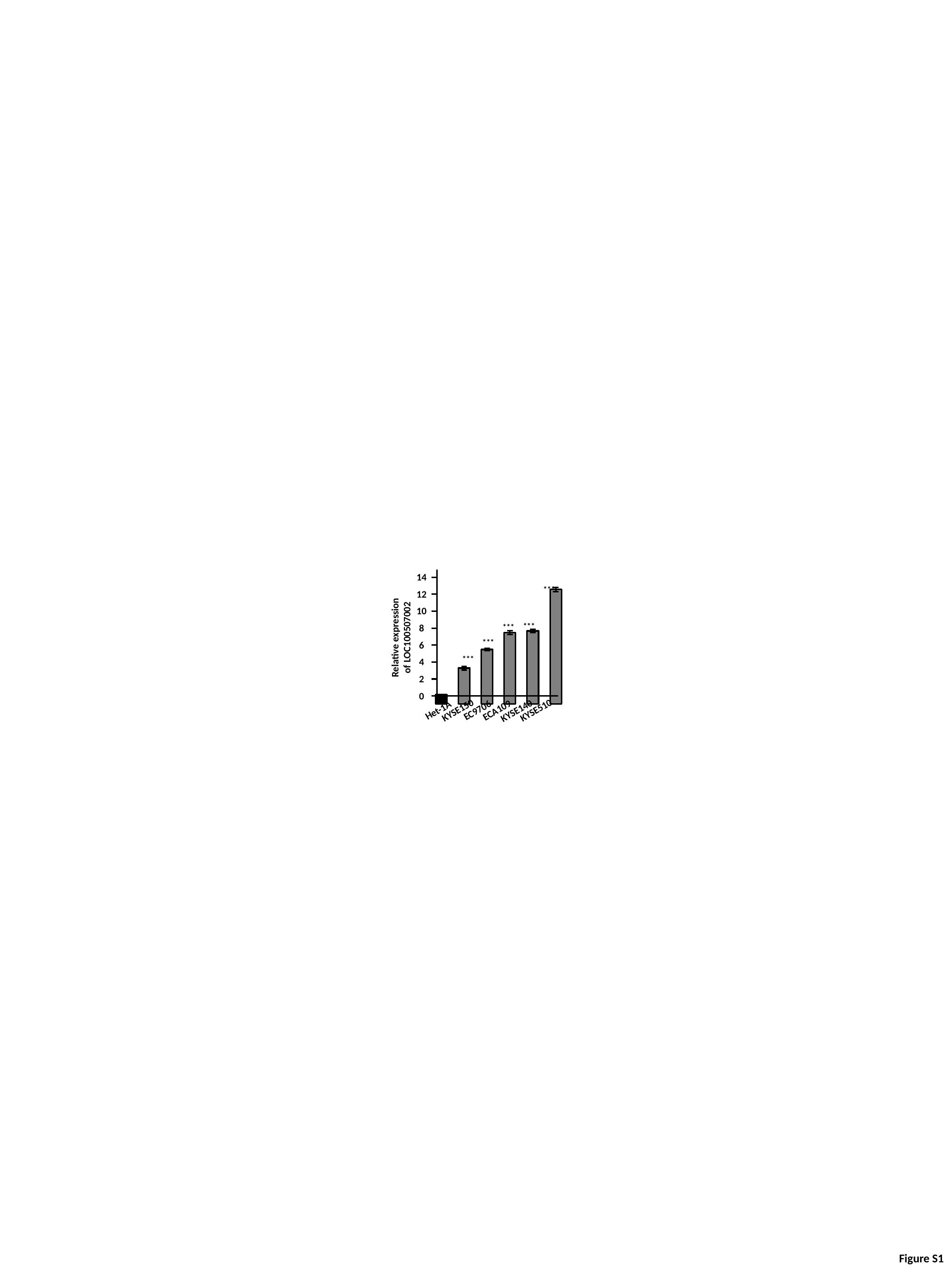

### Chart
| Category | |
|---|---|
| Het-1A | 1.0 |
| KYSE150 | 3.7163904720572325 |
| EC9706 | 5.645828240581022 |
| EC109 | 7.401855549668603 |
| KYSE140 | 7.572134081118752 |
| KYSE510 | 11.880458194306897 |14
6
4
2
0
Het-1A
KYSE150
EC9706
ECA109
KYSE510
KYSE140
***
***
***
***
Relative expression
of LOC100507002
***
12
10
8
Figure S1

## Slide 2
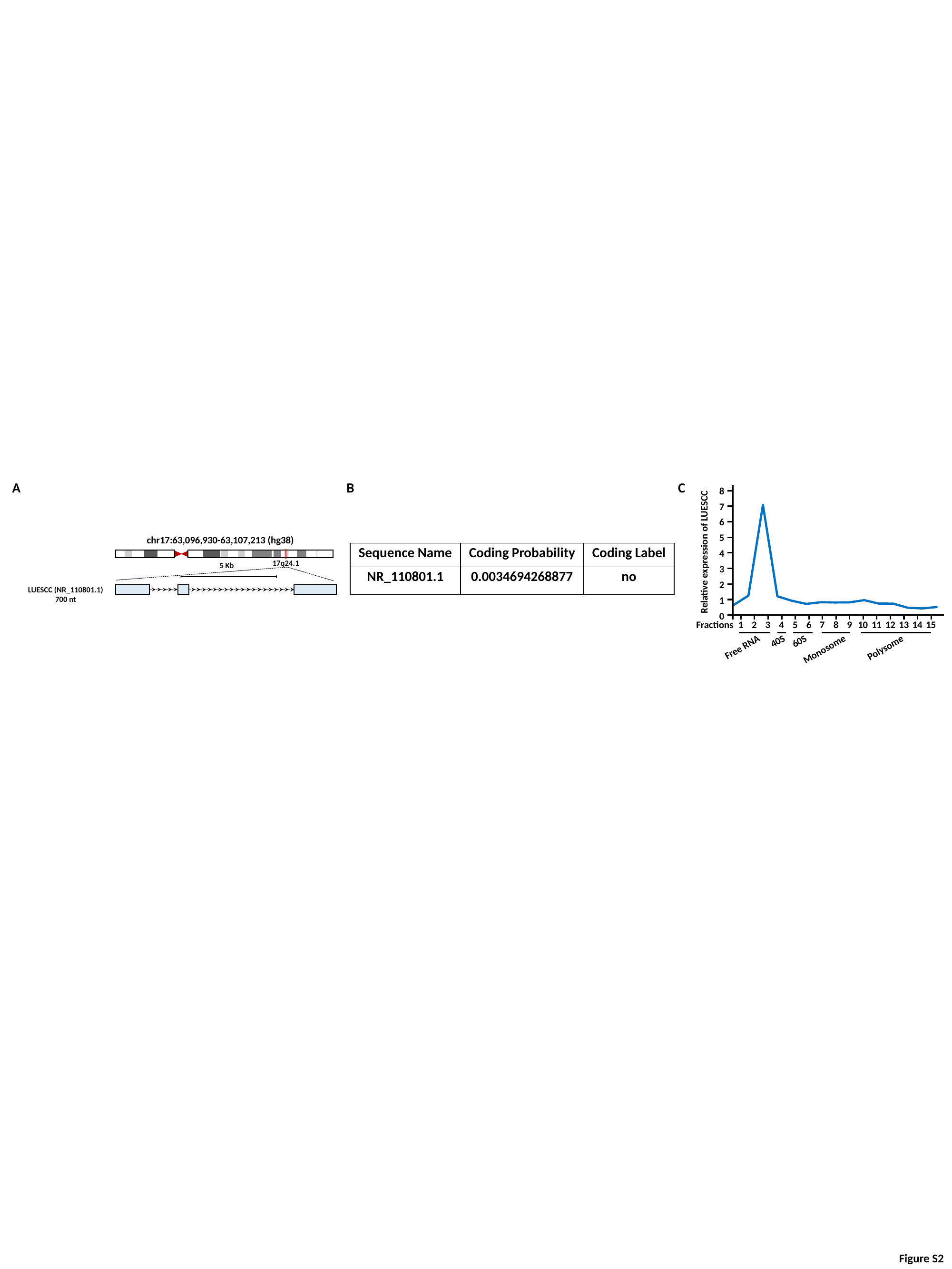

A
B
C
### Chart
| Category | |
|---|---|8
7
6
5
4
3
2
1
0
Fractions
1
2
3
4
5
6
7
8
9
10
11
12
13
14
15
40S
60S
Free RNA
Polysome
Monosome
Relative expression of LUESCC
chr17:63,096,930-63,107,213 (hg38)
17q24.1
5 Kb
LUESCC (NR_110801.1)
700 nt
| Sequence Name | Coding Probability | Coding Label |
| --- | --- | --- |
| NR\_110801.1 | 0.0034694268877 | no |
Figure S2

## Slide 3
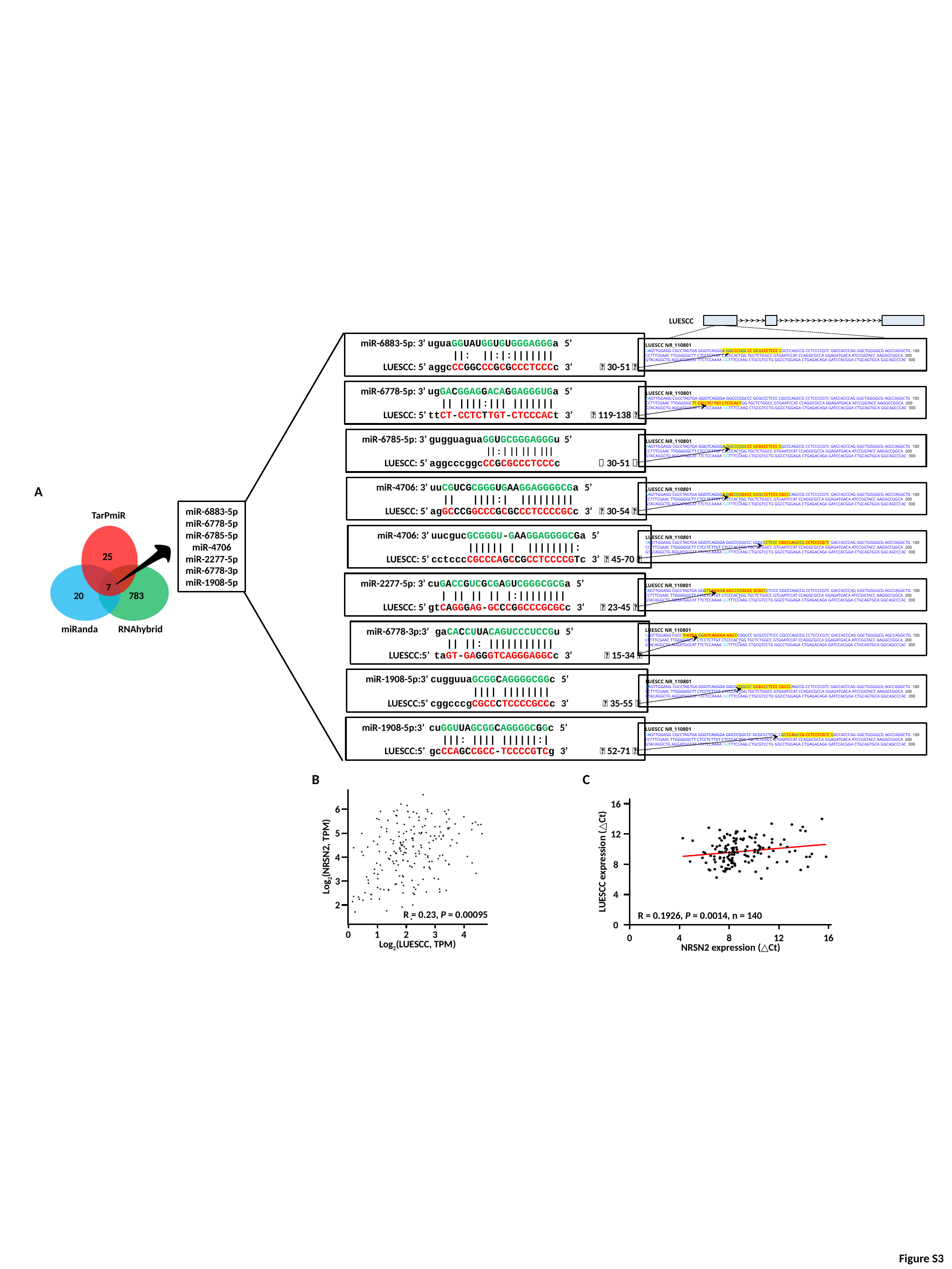

LUESCC
 miR-6883-5p: 3’ uguaGGUAUGGUGUGGGAGGGa 5’
 ||: ||:|:|||||||
 LUESCC: 5’ aggcCCGGCCCGCGCCCTCCCc 3’ （30-51）
LUESCC NR_110801
AAGTTGGAGG CGCCTAGTGA GGGTCAGGGA GGCCCGGCCC GCGCCCTCCC CGCCCAGCCG CCTCCCCGTC GACCACCCAG GGCTGGGGCG AGCCAGGCTG 100
CCTTTCGAAC TTGGGGGCTT CTCCTCTTGT CTCCCACTGG TGCTCTGGCC GTGAATCCAT CCAGGCGCCA GGAGATGACA ATCCGGTACC AAGGCCGGCA 200
GTACAGGCTG AGGATGGCAT TTCTCCAAAA GGTTTCCAAG CTGCGTCCTG GGCCTGGAGA CTGAGACAGA GATCCACGGA CTGCAGTGCA GGCAGCCCAC 300
……
 miR-6778-5p: 3’ ugGACGGAGGACAGGAGGGUGa 5’
 || ||||:||| |||||||
 LUESCC: 5’ ttCT-CCTCTTGT-CTCCCACt 3’ （119-138）
LUESCC NR_110801
AAGTTGGAGG CGCCTAGTGA GGGTCAGGGA GGCCCGGCCC GCGCCCTCCC CGCCCAGCCG CCTCCCCGTC GACCACCCAG GGCTGGGGCG AGCCAGGCTG 100
CCTTTCGAAC TTGGGGGCTT CTCCTCTTGT CTCCCACTGG TGCTCTGGCC GTGAATCCAT CCAGGCGCCA GGAGATGACA ATCCGGTACC AAGGCCGGCA 200
GTACAGGCTG AGGATGGCAT TTCTCCAAAA GGTTTCCAAG CTGCGTCCTG GGCCTGGAGA CTGAGACAGA GATCCACGGA CTGCAGTGCA GGCAGCCCAC 300
……
 miR-6785-5p: 3’ gugguaguaGGUGCGGGAGGGu 5’
 || : | || || | |||
 LUESCC: 5’ aggcccggcCCGCGCCCTCCCc （30-51）
LUESCC NR_110801
AAGTTGGAGG CGCCTAGTGA GGGTCAGGGA GGCCCGGCCC GCGCCCTCCC CGCCCAGCCG CCTCCCCGTC GACCACCCAG GGCTGGGGCG AGCCAGGCTG 100
CCTTTCGAAC TTGGGGGCTT CTCCTCTTGT CTCCCACTGG TGCTCTGGCC GTGAATCCAT CCAGGCGCCA GGAGATGACA ATCCGGTACC AAGGCCGGCA 200
GTACAGGCTG AGGATGGCAT TTCTCCAAAA GGTTTCCAAG CTGCGTCCTG GGCCTGGAGA CTGAGACAGA GATCCACGGA CTGCAGTGCA GGCAGCCCAC 300
……
 miR-4706: 3’ uuCGUCGCGGGUGAAGGAGGGGCGa 5’
 || ||||:| |||||||||
 LUESCC: 5’ agGCCCGGCCCGCGCCCTCCCCGCc 3’ （30-54）
A
LUESCC NR_110801
AAGTTGGAGG CGCCTAGTGA GGGTCAGGGA GGCCCGGCCC GCGCCCTCCC CGCCCAGCCG CCTCCCCGTC GACCACCCAG GGCTGGGGCG AGCCAGGCTG 100
CCTTTCGAAC TTGGGGGCTT CTCCTCTTGT CTCCCACTGG TGCTCTGGCC GTGAATCCAT CCAGGCGCCA GGAGATGACA ATCCGGTACC AAGGCCGGCA 200
GTACAGGCTG AGGATGGCAT TTCTCCAAAA GGTTTCCAAG CTGCGTCCTG GGCCTGGAGA CTGAGACAGA GATCCACGGA CTGCAGTGCA GGCAGCCCAC 300
……
miR-6883-5p
miR-6778-5p
miR-6785-5p
miR-4706
miR-2277-5p
miR-6778-3p
miR-1908-5p
TarPmiR
25
7
20
783
miRanda
RNAhybrid
 miR-4706: 3’ uucgucGCGGGU-GAAGGAGGGGCGa 5’
 |||||| | ||||||||:
 LUESCC: 5’ cctcccCGCCCAGCCGCCTCCCCGTc 3’ （45-70）
LUESCC NR_110801
AAGTTGGAGG CGCCTAGTGA GGGTCAGGGA GGCCCGGCCC GCGCCCTCCC CGCCCAGCCG CCTCCCCGTC GACCACCCAG GGCTGGGGCG AGCCAGGCTG 100
CCTTTCGAAC TTGGGGGCTT CTCCTCTTGT CTCCCACTGG TGCTCTGGCC GTGAATCCAT CCAGGCGCCA GGAGATGACA ATCCGGTACC AAGGCCGGCA 200
GTACAGGCTG AGGATGGCAT TTCTCCAAAA GGTTTCCAAG CTGCGTCCTG GGCCTGGAGA CTGAGACAGA GATCCACGGA CTGCAGTGCA GGCAGCCCAC 300
……
 miR-2277-5p: 3’ cuGACCGUCGCGAGUCGGGCGCGa 5’
 | || || || |:||||||||
 LUESCC: 5’ gtCAGGGAG-GCCCGGCCCGCGCc 3’ （23-45）
LUESCC NR_110801
AAGTTGGAGG CGCCTAGTGA GGGTCAGGGA GGCCCGGCCC GCGCCCTCCC CGCCCAGCCG CCTCCCCGTC GACCACCCAG GGCTGGGGCG AGCCAGGCTG 100
CCTTTCGAAC TTGGGGGCTT CTCCTCTTGT CTCCCACTGG TGCTCTGGCC GTGAATCCAT CCAGGCGCCA GGAGATGACA ATCCGGTACC AAGGCCGGCA 200
GTACAGGCTG AGGATGGCAT TTCTCCAAAA GGTTTCCAAG CTGCGTCCTG GGCCTGGAGA CTGAGACAGA GATCCACGGA CTGCAGTGCA GGCAGCCCAC 300
……
 miR-6778-3p:3’ gaCACCUUACAGUCCCUCCGu 5’
 || ||: |||||||||||
 LUESCC:5’ taGT-GAGGGTCAGGGAGGCc 3’ （15-34）
LUESCC NR_110801
AAGTTGGAGG CGCCTAGTGA GGGTCAGGGA GGCCCGGCCC GCGCCCTCCC CGCCCAGCCG CCTCCCCGTC GACCACCCAG GGCTGGGGCG AGCCAGGCTG 100
CCTTTCGAAC TTGGGGGCTT CTCCTCTTGT CTCCCACTGG TGCTCTGGCC GTGAATCCAT CCAGGCGCCA GGAGATGACA ATCCGGTACC AAGGCCGGCA 200
GTACAGGCTG AGGATGGCAT TTCTCCAAAA GGTTTCCAAG CTGCGTCCTG GGCCTGGAGA CTGAGACAGA GATCCACGGA CTGCAGTGCA GGCAGCCCAC 300
……
 miR-1908-5p:3’ cugguuaGCGGCAGGGGCGGc 5’
 |||| ||||||||
 LUESCC:5’ cggcccgCGCCCTCCCCGCCc 3’ （35-55）
LUESCC NR_110801
AAGTTGGAGG CGCCTAGTGA GGGTCAGGGA GGCCCGGCCC GCGCCCTCCC CGCCCAGCCG CCTCCCCGTC GACCACCCAG GGCTGGGGCG AGCCAGGCTG 100
CCTTTCGAAC TTGGGGGCTT CTCCTCTTGT CTCCCACTGG TGCTCTGGCC GTGAATCCAT CCAGGCGCCA GGAGATGACA ATCCGGTACC AAGGCCGGCA 200
GTACAGGCTG AGGATGGCAT TTCTCCAAAA GGTTTCCAAG CTGCGTCCTG GGCCTGGAGA CTGAGACAGA GATCCACGGA CTGCAGTGCA GGCAGCCCAC 300
……
 miR-1908-5p:3’ cuGGUUAGCGGCAGGGGCGGc 5’
 |||: |||| ||||||:|
 LUESCC:5’ gcCCAGCCGCC-TCCCCGTCg 3’ （52-71）
LUESCC NR_110801
AAGTTGGAGG CGCCTAGTGA GGGTCAGGGA GGCCCGGCCC GCGCCCTCCC CGCCCAGCCG CCTCCCCGTC GACCACCCAG GGCTGGGGCG AGCCAGGCTG 100
CCTTTCGAAC TTGGGGGCTT CTCCTCTTGT CTCCCACTGG TGCTCTGGCC GTGAATCCAT CCAGGCGCCA GGAGATGACA ATCCGGTACC AAGGCCGGCA 200
GTACAGGCTG AGGATGGCAT TTCTCCAAAA GGTTTCCAAG CTGCGTCCTG GGCCTGGAGA CTGAGACAGA GATCCACGGA CTGCAGTGCA GGCAGCCCAC 300
……
B
C
6
5
4
0
2
3
4
1
Log2(NRSN2, TPM)
Log2(LUESCC, TPM)
3
2
16
4
0
8
LUESCC expression (△Ct)
4
8
16
NRSN2 expression (△Ct)
0
12
12
R = 0.23, P = 0.00095
R = 0.1926, P = 0.0014, n = 140
Figure S3

## Slide 4
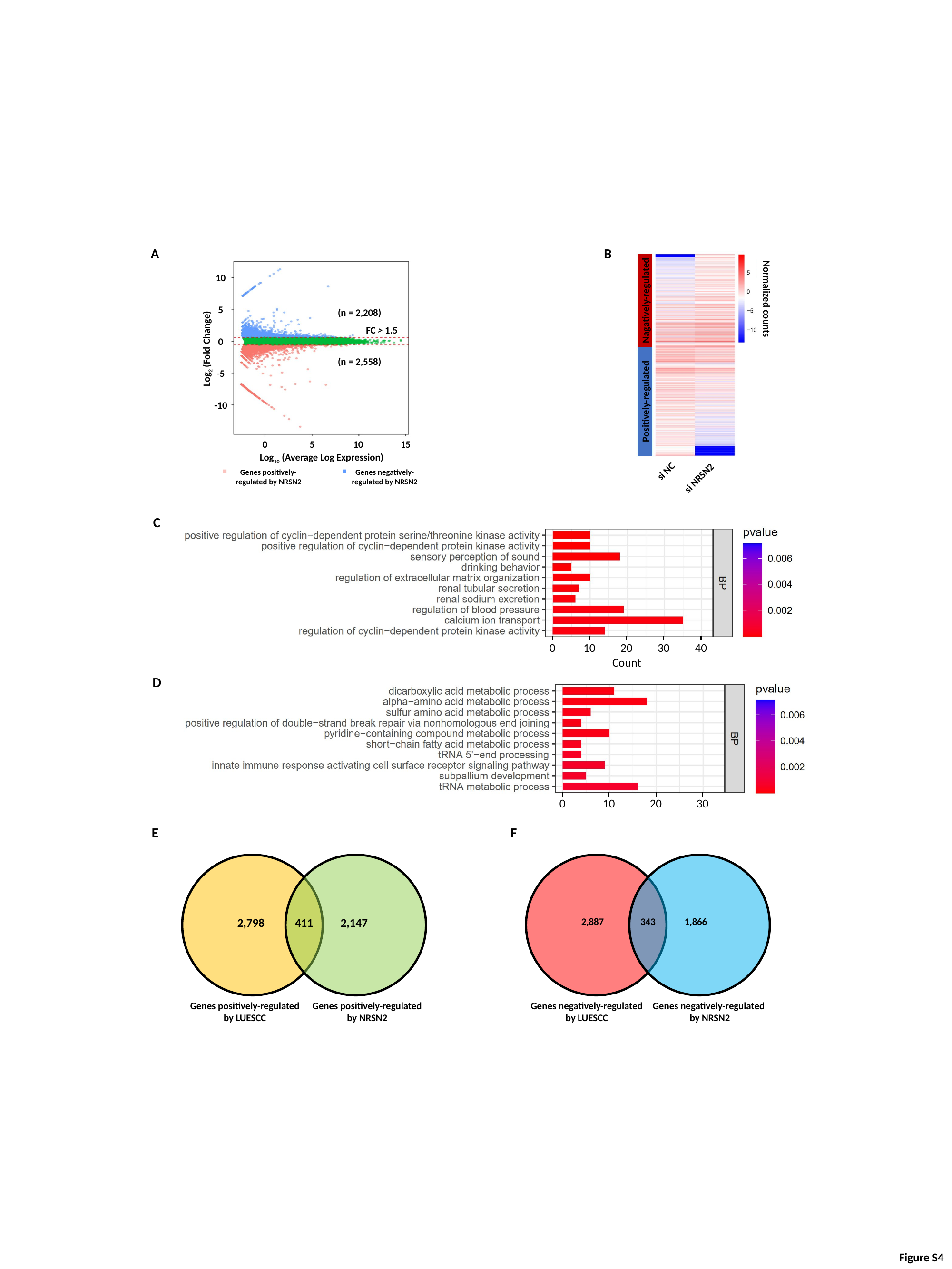

A
B
Normalized counts
Nagatively-regulated
Positively-regulated
si NC
si NRSN2
(n = 2,208)
FC > 1.5
(n = 2,558)
10
5
0
Log2 (Fold Change)
-5
-10
0
5
10
15
Log10 (Average Log Expression)
Genes positively-regulated by NRSN2
Genes negatively-regulated by NRSN2
C
0
10
20
30
40
Count
D
0
10
20
30
E
F
2,798
411
2,147
Genes positively-regulated
by LUESCC
Genes positively-regulated
by NRSN2
2,887
343
1,866
Genes negatively-regulated
by LUESCC
Genes negatively-regulated
by NRSN2
Figure S4

## Slide 5
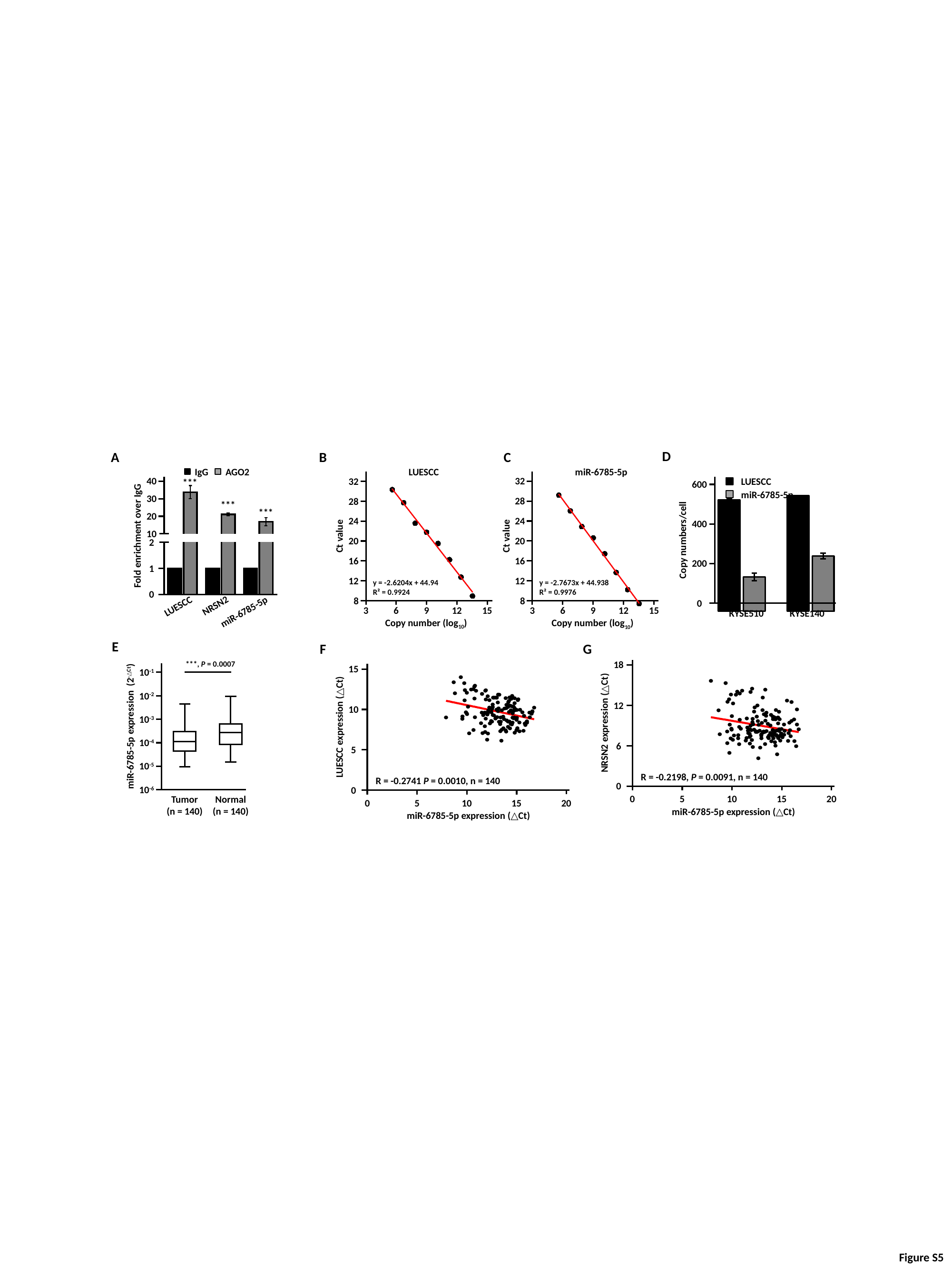

D
A
B
C
IgG
AGO2
40
***
30
***
***
20
10
Fold enrichment over IgG
2
1
0
NRSN2
LUESCC
miR-6785-5p
miR-6785-5p
32
28
24
20
16
8
Ct value
9
Copy number (log10)
3
6
y = -2.7673x + 44.938
R² = 0.9976
12
### Chart
| Category | |
|---|---|12
15
LUESCC
32
28
24
20
16
8
Ct value
12
Copy number (log10)
3
9
y = -2.6204x + 44.94
R² = 0.9924
12
### Chart
| Category | |
|---|---|15
6
LUESCC
miR-6785-5p
600
Copy numbers/cell
0
KYSE510
400
200
KYSE140
### Chart
| Category | | |
|---|---|---|E
F
G
***, P = 0.0007
10-1
10-2
10-3
miR-6785-5p expression (2-△Ct)
10-4
10-6
Tumor
(n = 140)
Normal
(n = 140)
10-5
18
6
0
12
NRSN2 expression (△Ct)
5
10
20
miR-6785-5p expression (△Ct)
0
15
R = -0.2198, P = 0.0091, n = 140
15
5
0
10
LUESCC expression (△Ct)
5
10
20
miR-6785-5p expression (△Ct)
0
15
R = -0.2741 P = 0.0010, n = 140
Figure S5
