# Supplementary material for: LncRNA LUESCC promotes esophageal squamous cell carcinoma by targeting the miR-6785-5p/NRSN2 axis
Source: Cell Mol Life Sci. 2024 Mar 8;81(1):121. doi: 10.1007/s00018-024-05172-9 (PMC10924007; doi:10.1007/s00018-024-05172-9)
Supplement: Supplementary file 8 — Supplementary file8 (DOCX 18 KB) [file 18_2024_5172_MOESM8_ESM.docx]

**Supplementary information**

**Additional file 1: Table S1.** Primers and oligonucleotides used in this study.

**Additional file 2: Table S2.** The correlation between the expression of dysregulated lncRNAs and the prognosis in ESCC patients.

**Additional file 3: Table S3.** The correlation between the clinicopathologic characteristics and LOC100507002 expression in ESCC.

**Additional file 4: Table S4.** MiRNAs that are predicted to bind to LUESCC and its target genes.

**Additional file 5: Table S5.** The correlation between the expression of genes positively-regulated by LUESCC and prognosis.

**Additional file 6: Table S6.** Genes that are regulated, both positively (sheet 1) and negatively (sheet 2), by NRSN2 detected by RNA-seq analysis (FC > 1.5) as well as genes that are commonly regulated by LUESCC and NRSN2 (sheet 3).

**Additional file 7: Fig. S1** The expression of LOC100507002 in ESCC cell lines (KYSE140, KYSE150, KYSE510, EC109, and EC9706) and normal esophageal epithelial cell line (Het-1A) is shown.

**Additional file 8: Fig. S2** **LUESCC has no coding potential.**

**A** Schematic diagram of the chromosomal location of LUESCC. **B** Coding potential for LUESCC was predicted by CPAT website tools. **C** KYSE510 cells were subjected to polysome profiling, and the resultant fractions were applied to RNA exaction and RT-qPCR analysis to examine the expression of LUESCC. Fractions 1 to 3: free RNA (unbound with ribosome); Fraction 4: 40S (40S ribosomal subunit); Fractions 5 and 6: 60S (60S ribosomal subunit); Fractions 7 to 9: monosome; Fractions 10 to 15: polysome.

**Additional file 9: Fig. S3** **LUESCC acts as a miRNA sponge for miR-6785-5p to regulate the expression of NRSN2.**

**A** Three different miRNA prediction algorithms as indicted were used to predict potential miRNAs that can bind to LUESCC, and the highly confident miRNAs predicted were then overlapped, leading to seven miRNAs in common, including miR-6883-5p, miR-6778-5p, miR-6785-5p, miR-4706, miR-2277-5p, miR-6778-3p, and miR-1908-5p. The binding sites for the predicted miRNAs in LUESCC are highlighted (yellow). **B, C** The correlation between the expression of LUESCC and NRSN2 in TCGA database (B) and in-house cohort (C).

**Additional file 10: Fig. S4 LUESCC and NRSN2 regulates the expression of genes involved in calcium channel, calcineurin/NFAT1, and TGF-β signaling pathway.**

**A** KYSE510 cells transfected with negative control siRNA (si NC) or siRNA specifically targeting NRSN2 (si NRSN2) for three days were subjected to RNA-seq analysis, and differentially expressed genes are presented by MA plot. Blue and red dots represent upregulated and downregulated genes, respectively (FC > 1.5). **B** The expression of differentially expressed genes as described in (A) is represented by heat map. **C, D** Gene ontology analysis for genes that are positively- (C) or negatively- (D) regulated by NRSN2 as described in (A) is shown. **E, F** Genes positively- or negatively-regulated by LUESCC and NRSN2 are presented by Venn plot (FC > 1.5).

**Additional file 11: Fig. S5 LUESCC acts as a miRNA sponge for miR-6785-5p to regulate the expression of NRSN2.**

**A** AGO2-CLASH assay was performed to examine the interaction of LUESCC and NRSN2 with miR-6785-5p. **B, C** The standard curves for copy number analysis of LUESCC (B) and miR-6785-5p (C). **D** The copy number of LUESCC and miR-6785-5p in KYSE510 and KYSE140 cells measured by RT-qPCR analysis. **E** The expression of miR-6785-5p in a cohort of ESCC tumor samples (n = 140) and normal samples (n = 140) in house. **F, G** The correlation between the expression of miR-6785-5p and LUESCC (F) or NRSN2 (G).
